# Boundary layer flow past a stretching/shrinking surface beneath an external uniform shear flow with a convective surface boundary condition in a nanofluid

**DOI:** 10.1186/1556-276X-6-314

**Published:** 2011-04-07

**Authors:** Nor Azizah Yacob, Anuar Ishak, Ioan Pop, Kuppalapalle Vajravelu

**Affiliations:** 1 Faculty of Computer and Mathematical Sciences, Universiti Teknologi MARA Pahang, 26400 Bandar Tun Razak Jengka, Pahang, Malaysia; 2 Centre for Modelling & Data Analysis, School of Mathematical Sciences, Faculty of Science and Technology, Universiti Kebangsaan Malaysia, 43600 UKM Bangi, Selangor, Malaysia; 3 Faculty of Mathematics, University of Cluj, R-3400 Cluj, CP 253, Romania; 4 Department of Mathematics, University of Central Florida, Orlando, FL 32816, USA

## Abstract

The problem of a steady boundary layer shear flow over a stretching/shrinking sheet in a nanofluid is studied numerically. The governing partial differential equations are transformed into ordinary differential equations using a similarity transformation, before being solved numerically by a Runge-Kutta-Fehlberg method with shooting technique. Two types of nanofluids, namely, Cu-water and Ag-water are used. The effects of nanoparticle volume fraction, the type of nanoparticles, the convective parameter, and the thermal conductivity on the heat transfer characteristics are discussed. It is found that the heat transfer rate at the surface increases with increasing nanoparticle volume fraction while it decreases with the convective parameter. Moreover, the heat transfer rate at the surface of Cu-water nanofluid is higher than that at the surface of Ag-water nanofluid even though the thermal conductivity of Ag is higher than that of Cu.

## Introduction

Blasius [1] was the first who studied the steady boundary layer flow over a fixed flat plate with uniform free stream. Howarth [2] solved the Blasius problem numerically. Since then, many researchers have investigated the similar problem with various physical aspects [3-6]. In contrast to the Blasius problem, Sakiadis [7] introduced the boundary layer flow induced by a moving plate in a quiescent ambient fluid. Tsou et al. [8] studied the flow and temperature fields in the boundary layer on a continuous moving surface, both analytically and experimentally and verified the results obtained in [7]. Crane [9] extended this concept to a stretching plate in a quiescent fluid with a stretching velocity that varies with the distance from a fixed point and presented an exact analytic solution. Different from the above studies, Miklavčič and Wang [10] examined the flow due to a shrinking sheet where the velocity moves toward a fixed point. Fang [11] studied the boundary layer flow over a shrinking sheet with a power-law velocity, and obtained exact solutions for some values of the parameters.

It is well known that Choi [12] was the first to introduce the term "nanofluid" that represents the fluid in which nano-scale particles are suspended in the base fluid with low thermal conductivity such as water, ethylene glycol, oils, etc. [13]. In recent years, the concept of nanofluid has been proposed as a route for surpassing the performance of heat transfer rate in liquids currently available. The materials with sizes of nanometers possess unique physical and chemical properties [14]. They can flow smoothly through microchannels without clogging them because they are small enough to behave similar to liquid molecules [15]. This fact has attracted many researchers such as [16-27] to investigate the heat transfer characteristics in nanofluids, and they found that in the presence of the nanoparticles in the fluids, the effective thermal conductivity of the fluid increases appreciably and consequently enhances the heat transfer characteristics. An excellent collection of articles on this topic can be found in [28-33], and in the book by Das et al. [14].

It is worth mentioning that while modeling the boundary layer flow and heat transfer of stretching/shrinking surfaces, the boundary conditions that are usually applied are either a specified surface temperature or a specified surface heat flux. However, there are boundary layer flow and heat transfer problems in which the surface heat transfer depends on the surface temperature. Perhaps the simplest case of this is when there is a linear relation between the surface heat transfer and surface temperature. This situation arises in conjugate heat transfer problems (see, for example, [34]), and when there is Newtonian heating of the convective fluid from the surface; the latter case was discussed in detail by Merkin [35]. The situation with Newtonian heating arises in what is usually termed as conjugate convective flow, where the heat is supplied to the convective fluid through a bounding surface with a finite heat capacity. This results in the heat transfer rate through the surface being proportional to the local difference in the temperature with the ambient conditions. This configuration of Newtonian heating occurs in many important engineering devices, for example, in heat exchangers, where the conduction in a solid tube wall is greatly influenced by the convection in the fluid flowing over it. On the other hand, most recently, heat transfer problems for boundary layer flow concerning with a convective boundary condition were investigated by Aziz [36], Makinde and Aziz [37], Ishak [38], and Magyari [39] for the Blasius flow. Similar analysis was applied to the Blasius and Sakiadis flows with radiation effects by Bataller [4]. Yao et al. [40] have very recently investigated the heat transfer of a viscous fluid flow over a permeable stretching/shrinking sheet with a convective boundary condition. Magyari and Weidman [41] investigated the heat transfer characteristics on a semi-infinite flat plate due to a uniform shear flow, both for the prescribed surface temperature and prescribed surface heat flux. It is worth pointing out that a uniform shear flow is driven by a viscous outer flow of rotational velocity whereas the classical Blasius flow is driven over the plate by an inviscid outer flow of irrotational velocity.

The objective of this study is to extend the study of Magyari and Weidman [41] to a stretching/shrinking surface with a convective boundary condition immersed in a nanofluid, that is, to study the steady boundary layer shear flow over a stretching/shrinking surface beneath an external uniform shear flow with a convective surface boundary condition in a nanofluid. This problem is relevant to several practical applications in the field of metallurgy, chemical engineering, etc. A number of technical processes concerning polymers involve the cooling of continuous strips or filaments by drawing them through a quiescent fluid. In these cases, the properties of the final product depend to a great extent on the rate of cooling, which is governed by the structure of the boundary layer near the stretching/shrinking surface. The governing partial differential equations are transformed into ordinary differential equations using a similarity transformation, before being solved numerically by the Runge-Kutta-Fehlberg method with shooting technique.

## Mathematical formulation

**Figure 1:**
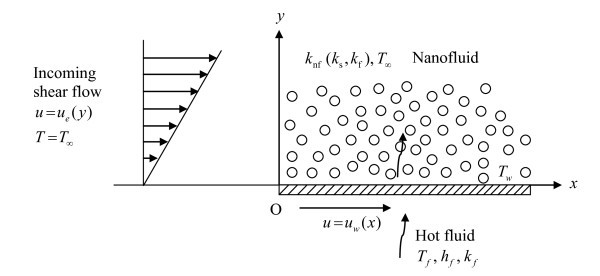
**Physical model and the coordinate system**.

Consider a two-dimensional steady boundary layer shear flow over a stretching/shrinking sheet in a laminar and incompressible nanofluid of ambient temperature *T_∞_*. The fluid is a water-based nanofluid containing two type of nanoparticles, either Cu (copper) or Ag (silver). The nanoparticles are assumed to have a uniform shape and size. Moreover, it is assumed that both the fluid phase and nanoparticles are in thermal equilibrium state. Figure 1 describes the physical model and the coordinate system, where the *x *and *y *axes are measured along the surface of the sheet and normal to it, respectively. Following Magyari and Weidman [41], it is assumed that the velocity of the moving stretching/shrinking sheet is *u*_w_(*x*) = *U*_w_(*x*/*L*)^1/3 ^and the velocity outside the boundary layer (potential flow) is *u*_e_(*y*) = β*y*, where β is the constant strain rate. We also assume that the bottom surface of the stretching/shrinking surface is heated by convection from a base (water) fluid at temperature *T*_f_, which provides a heat transfer coefficient *h*_f _(see [36]). Under the boundary layer approximations, the basic equations are (see [17,42]),(1)(2)(3)

Further, we assume that the sheet surface temperature is maintained by convective heat transfer at a constant temperature *T*_w _(see [36]). Thus, the boundary conditions of Equations 1-3 are(4)

where *L *is the characteristic length of the stretching/shrinking surface. The properties of nanofluids are defined as follows (see [20]):(5)

Following Magyari and Weidman [41] and Aziz [36], we look for a similarity solution of Equations 1-3 of the form:(6)

where ν_f _is the kinematic viscosity of the base (water) fluid, and *ψ *is the stream function, which is defined as *u*= ∂*ψ*/∂*y *and *v *= –∂*ψ*/∂*x*, which automatically satisfies Equation 1. A simple analysis shows that *L *= (ν_f_/β)^1/2^. Substituting (6) into Equations 2 and 3, we obtain the following ordinary differential equations:(7)(8)

subject to the boundary conditions(9)

where primes denote differentiation with respect to η, and λ = *U*_w_/(βν_f_)^1/2 ^is the stretching/shrinking parameter, and *γ *is given by(10)

For the thermal equation (8) to have a similarity solution, the quantity *γ *must be a constant and not a function of *x *as in Equation 10. This condition can be met if *h*_f _is proportional to (*x*/*L*)^-1/3^. We, therefore, assume(11)

where *c *is a constant. Thus, we have(12)

with *γ *defined by Equation 12, the solutions of Equations 7-9 yield the similarity solutions. However, with *γ *defined by Equation 10, the generated solutions are local similarity solutions. We notice that the solution of Equations 7 and 8 approaches the solution for the constant surface temperature as *γ *→ ∞. This can be seen from the boundary conditions (9), which gives θ(0) = 1 as *γ *→ ∞. Further, it is worth mentioning that Equations 7 and 8 reduce to those of Magyari and Weidman [41] when φ = 0 (regular fluid) and λ = 0 (fixed surface).

The quantities of interest are the skin friction coefficient *C*_f _and the local Nusselt number *Nu_x_*, which represents the heat transfer rate at the surface, and they can be shown to be given in dimensionless form as(13)

## Results and discussion

**Figure 2:**
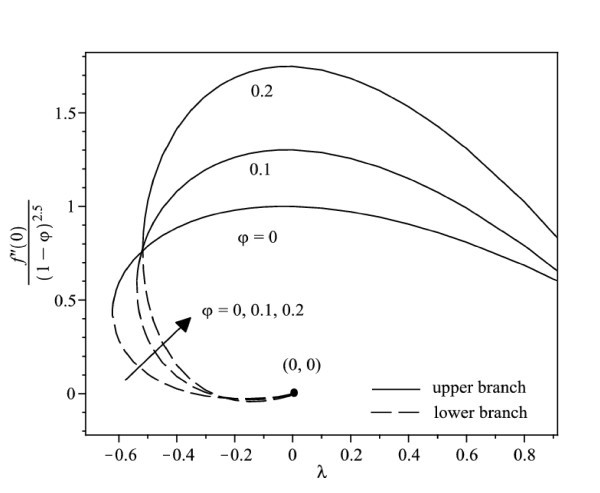
**Variation of the skin friction coefficient with λ for different values of φ when *γ *= 0.5 for Ag-water nanofluid**.

**Figure 3:**
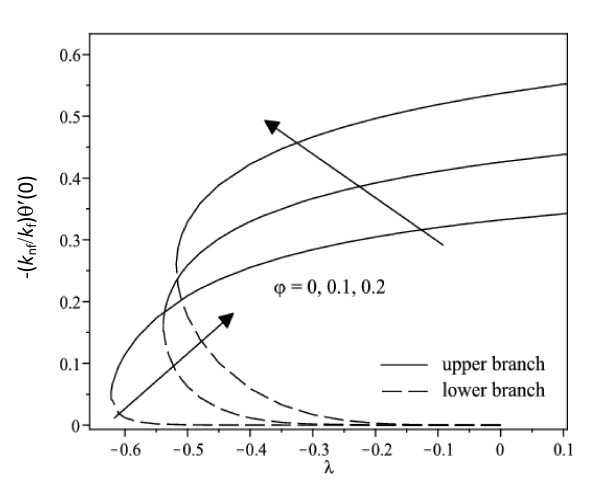
**Variation of the local Nusselt number with λ for different values of φ when *γ *= 0.5 for Ag-water nanofluid**.

**Figure 4:**
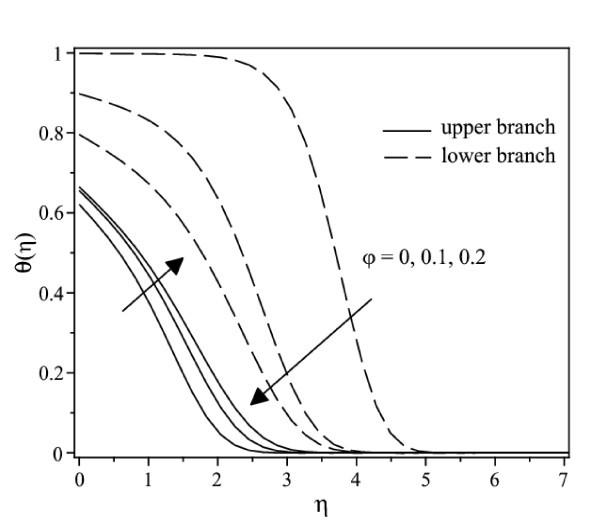
**Temperature profiles for Cu-water nanofluid when *γ *= 0.5 and λ = -0.53 for different values of φ**.

**Figure 5:**
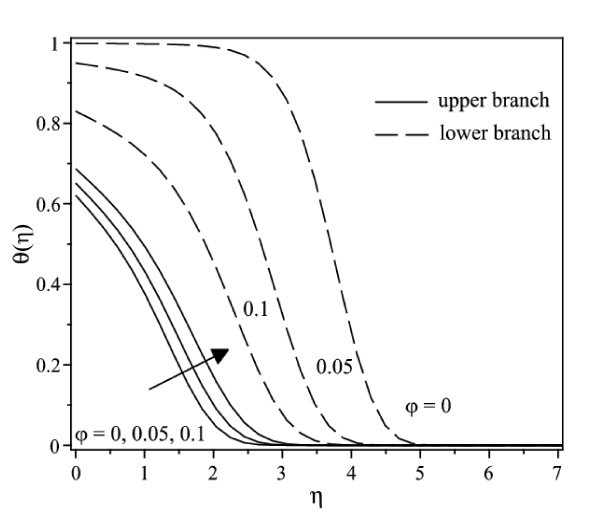
**Temperature profiles for Ag-water nanofluid when *γ *= 0.5 and λ = -0.53 for different values of φ**.

The nonlinear ordinary differential equations (7) and (8) subject to the boundary conditions (9) were solved numerically by the Runge-Kutta-Fehlberg method with shooting technique. We consider two different types of nanoparticles, namely, Cu and Ag with water as the base fluid. Table 1 shows the thermophysical properties of water and the elements Cu and Ag. The Prandtl number of the base fluid (water) is kept constant at 6.2. It is worth mentioning that this study reduces to those of a viscous or regular fluid when φ = 0. Figure 2 shows the variation of the skin friction coefficient (1/(1-*φ*)^2.5^)*f"*(0) with λ of Ag-water nanofluid when *γ *= 0.5 for different nanoparticle volume fraction φ, while the respective local Nusselt number -(*k*_nf_/*k*_f_) θ' (0) is displayed in Figure 3. It can be seen that for a particular value of λ, the skin friction coefficient and the local Nusselt number increase with increasing φ. Dual solutions are found to exist when λ < 0 (shrinking case) as displayed in Figures 2 and 3. Moreover, the solution can be obtained up to a critical value of λ (say λ_c_), and |λ_c_| decreases with increasing φ. The similar pattern is observed for Cu-water nanofluid, which is not presented here, for the sake of brevity. It is observed that, the solution is unique for λ ≥ 0, dual solutions exist for λ_c _< λ < 0, and no solution for λ <*λ*_c_. The values of λ_c _for Ag-water nanofluid and Cu-water nanofluid for different values of φ are presented in Table 2. It is seen that for φ = 0.1 and φ = 0.2, the value of |λ_c_| for Cu-water nanofluid is greater than those of Ag-water nanofluid. The temperature profiles of Ag-water and Cu-water nanofluids for different values of φ when *γ *= 0.5 and λ = -0.53 are presented in Figures 4 and 5, respectively. These profiles show that, there exist two different profiles satisfying the far field boundary condition (9) asymptotically, thus supporting the dual nature of the solutions presented in Figures 2 and 3. Both Figures 4 and 5 show that the boundary layer thickness is higher for the second solution compared to the first solution, which in turn produces higher surface temperature θ(0) for the former.

**Table 1:** Thermophysical properties of water and the elements Cu and Ag

Physical Properties	Fluid Phase (Water)	Cu	Ag
*C*_p _(J/kgK)	4179	385	235
ρ (KG/m^3^)	997.1	8933	10500
*k *(W/mK)	0.613	400	429
α × 10^7 ^(m^2^/s)	1.47	1163.1	1738.6

**Table 2:** Values of λ_c _for Cu-water and Ag-water nanofluids

φ	λ_c_	
	Cu	Ag
0	-0.62228	-0.62228
0.1	-0.55512	-0.53870
0.2	-0.53929	-0.51800

**Figure 6:**
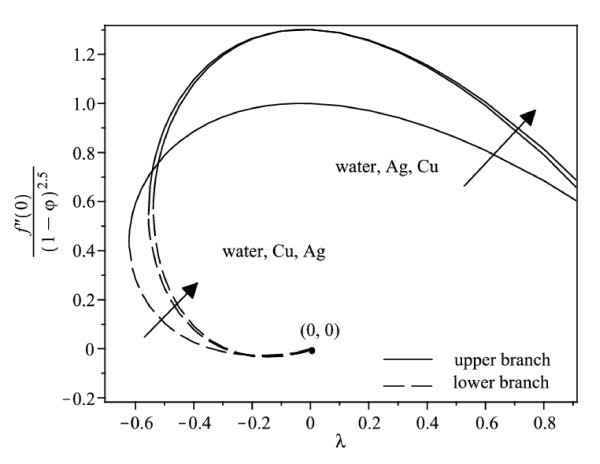
**Variation of the skin friction coefficient with λ when *γ *= 0.5 and φ = 0.1 for different nanofluids and water**.

**Figure 7:**
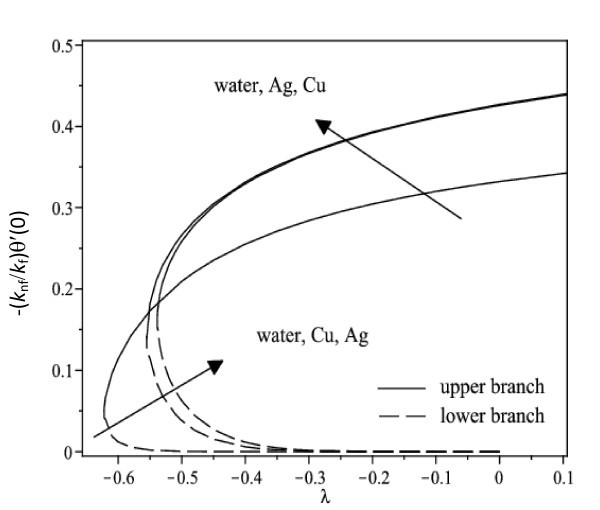
**Variation of the local Nusselt number with λ when *γ *= 0.5 and φ = 0.1 for different nanofluids and water**.

**Figure 8:**
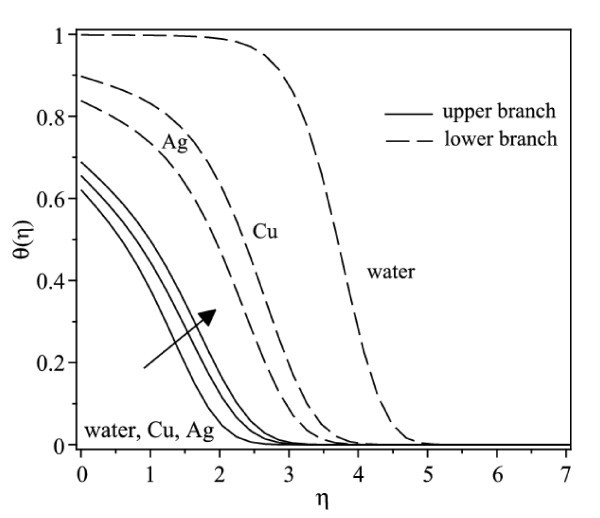
**Temperature profiles for different nanofluids and water when *γ *= 0.5, λ = -0.53, and φ = 0.1**.

Figure 6 displays the variation of the skin friction coefficient (1/(1-*φ*)^2.5^)*f"*(0) with λ when *γ *= 0.5 for water, Cu-water and Ag-water nanofluids, while the respective local Nusselt number -(*k*_nf_/*k*_f_)*θ'*(0) is shown in Figure 7. In general, for a particular value of λ, the skin friction coefficient of Cu-water nanofluid is higher than that of Ag-water nanofluid and that of water for the upper branch solutions, while the skin friction coefficient of Ag-water nanofluid is higher than that of Cu-water nanofluid and that of water for the lower branch solutions. Further, Figure 7 shows that Cu-water nanofluid has the highest local Nusselt number compared with Ag-water nanofluid and water for the upper branch solutions. From this observation, the heat transfer rate at the surface of Cu-water nanofluid is higher than that of Ag-water nanofluid even though Ag has higher thermal conductivity than the thermal conductivity of Cu as presented in Table 1. However, the difference in heat transfer rate at the surface is small. On the other hand, Ag-water nanofluid has the highest local Nusselt number compared with Cu-water nanofluid and water for the lower branch solutions. The corresponding temperature profiles that support the results obtained in Figure 7 when λ = -0.53 is shown in Figure 8.

It is observed from Figures 2, 3, 6, and 7 that the skin friction coefficient and the local Nusselt number are more influenced by the nanoparticle volume fraction than the types of nanoparticles. This observation is in agreement with those obtained by Oztop and Abu-Nada [20] and Abu-Nada and Oztop [43]. In addition, water has the lowest skin friction coefficient and local Nusselt number compared with Cu-water and Ag-water nanofluids. The range of λ for which the solution exists is wider for water compared with the others.

**Figure 9:**
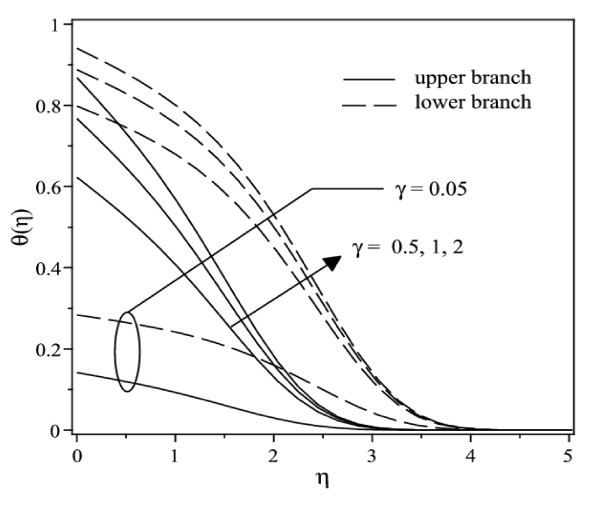
**Temperature profiles for different values of *γ *when λ = -0.5 and φ = 0.2 for Ag-water nanofluid**.

The temperature profiles of Ag-water nanofluid for different values of convective parameter *γ *when *φ *= 0.2 is presented in Figure 9. It is observed that the surface temperature increases with an increase in *γ *for both solution branches, and in consequence, decreases the local Nusselt number. It can be seen that from the convective boundary conditions (9), the value of θ(0) approaches 1, as *γ *→ ∞. Further, the convective parameter *γ *as well as the Prandtl number Pr has no influence on the flow field, which is clear from Equations 7-9. Finally, it is worth mentioning that all the velocity and temperature profiles presented in Figures 4, 5, 7, 8, and 9 satisfy the far-field boundary conditions (9) asymptotically, thus supporting the validity of the numerical results obtained.

## Conclusions

The problem of a steady boundary layer shear flow over a stretching/shrinking sheet in a nanofluid was studied numerically. The governing partial differential equations were transformed into ordinary differential equations by a similarity transformation, before being solved numerically using the Runge-Kutta-Fehlberg method with shooting technique. We considered two types of nanofluids, namely, Cu-water and Ag-water. It was found that the heat transfer rate at the surface increases with increasing nanoparticle volume fraction while it decreases with the convective parameter. The variations of the skin friction coefficient and the heat transfer rate at the surface are more influenced by the nanoparticle volume fraction than the types of the nanofluids. Moreover, the heat transfer rate at the surface of Cu-water nanofluid is higher than that of the Ag-water nanofluid even though Ag has higher thermal conductivity than that of Cu.

## Abbreviations

**List of symbols**: *c*: Constant; *C*_f_: Skin friction coefficient; *C*_p_: Specific heat at constant pressure; *f*: Dimensionless stream function; *h*_f_: Heat transfer coefficient; *k*: Thermal conductivity; *L*: Reference length; *Nu_x_*: Local Nusselt number; Pr: Prandtl number; *q*_w_: Surface heat flux; *T*: Fluid temperature; *T*_f_: Temperature of the hot fluid; *T*_w_: Surface temperature; *T_∞_*: Ambient temperature; *u*, *v*: Velocity components along the *x *and *y*-directions, respectively; *u_e_*(*y*): Free stream velocity; *u*_w_(*x*): Stretching/shrinking velocity; *U*_w_: Reference stretching/shrinking velocity; *x*, *y*: Cartesian coordinates along the surface and normal to it, respectively; **Greek symbols**: α: Thermal diffusivity; β: Constant strain rate; γ: Convective parameter; η: Similarity variable; θ: Dimensionless temperature; λ: Stretching/shrinking parameter; μ: Dynamic viscosity; ν: Kinematic viscosity; ρ: Fluid density; φ: Nanoparticle volume fraction; ψ: Stream function; τ_*w*_: Wall shear stress; **Subscripts**; f: Fluid; nf: Nanofluid; s: Solid.

## Competing interests

The authors declare that they have no competing interests.

## Authors' contributions

NAY and AI performed the numerical analysis and wrote the manuscript. IP carried out the literature review and co-wrote the manuscript. KV helped to draft the manuscript. All authors read and approved the final manuscript.
